# Mediastinal Germ Cell Tumor with Testicular Microlithiasis

**DOI:** 10.7759/cureus.12761

**Published:** 2021-01-18

**Authors:** Nasser Mulla

**Affiliations:** 1 Department of Internal Medicine, College of Medicine, Taibah University, Madinah, SAU

**Keywords:** extragonadal germ cell tumor, mediastinal tumor, testicular microlithiasis

## Abstract

Testicular microlithiasis (TM) is a condition in which punctate calcifications are present in the testicle. A case of a 29-year-old Saudi male who developed a cough, hemoptysis, and shortness of breath is presented in this report. A computed tomography (CT) scan of the chest revealed a large mediastinal mass. CT scans of the abdomen and pelvis were normal. Testicular ultrasonography (US) showed multiple, bilateral punctate echoes that are characteristic of TM. No primary testicular tumor was detected. Transthoracic needle biopsy of the mediastinal tumor was consistent with a mixed germ cell tumor. This is the seventh case of extragonadal germ cell tumor with TM.

## Introduction

Testicular microlithiasis (TM) is an entity of unknown etiology that is characterized by the presence of intratubular calcifications [1]. The prevalence varies from 0.6% to 9.0% [2,3]. On ultrasound (US), it appears as multiple, uniform, non-shadowing echogenic foci in the testis [4]. While there is a known association of TM with testicular malignancy [5], its association with extragonadal germ cell tumor is rare [6]. The author reports a case of extragonadal (mediastinal) germ cell tumor (non-seminoma) and TM.

## Case presentation

Patient information and clinical findings

In March 2020, a 29-year-old male presented to the emergency department complaining of neck pain, cough, hemoptysis, and shortness of breath. He had no significant medical history and was not taking any medications. His family history was unremarkable. He was single and sexually inactive. Local testicular examination was unremarkable.

**Figure 1:**
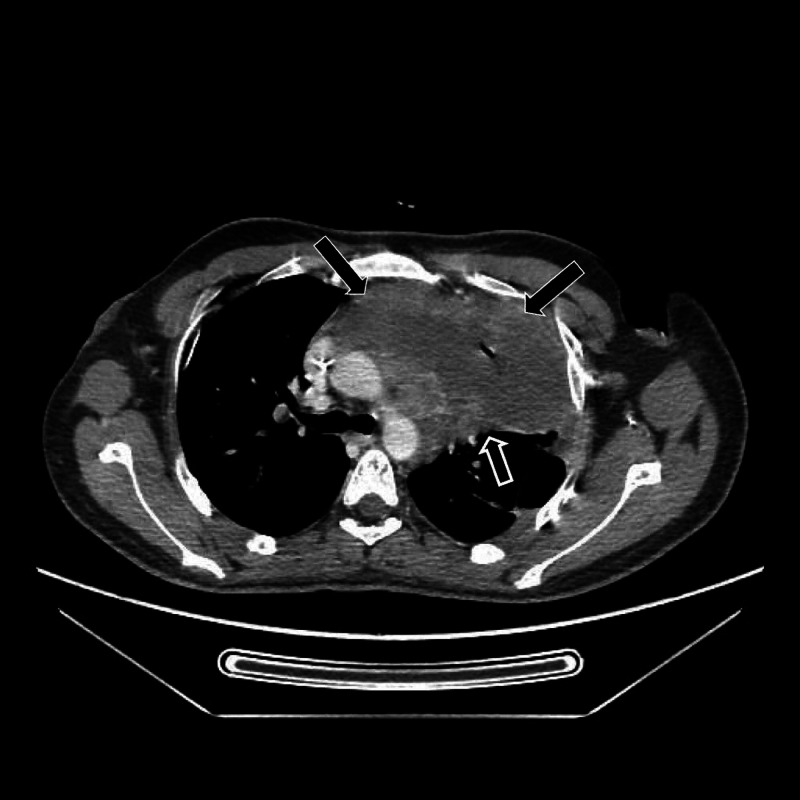
Computed tomography (CT) of the chest showing a large mass in the superior mediastinum.

Diagnostic assessment: A computed tomography (CT) of the chest demonstrated a large soft tissue mass in the anterior mediastinum (Figure 1).

**Figure 2:**
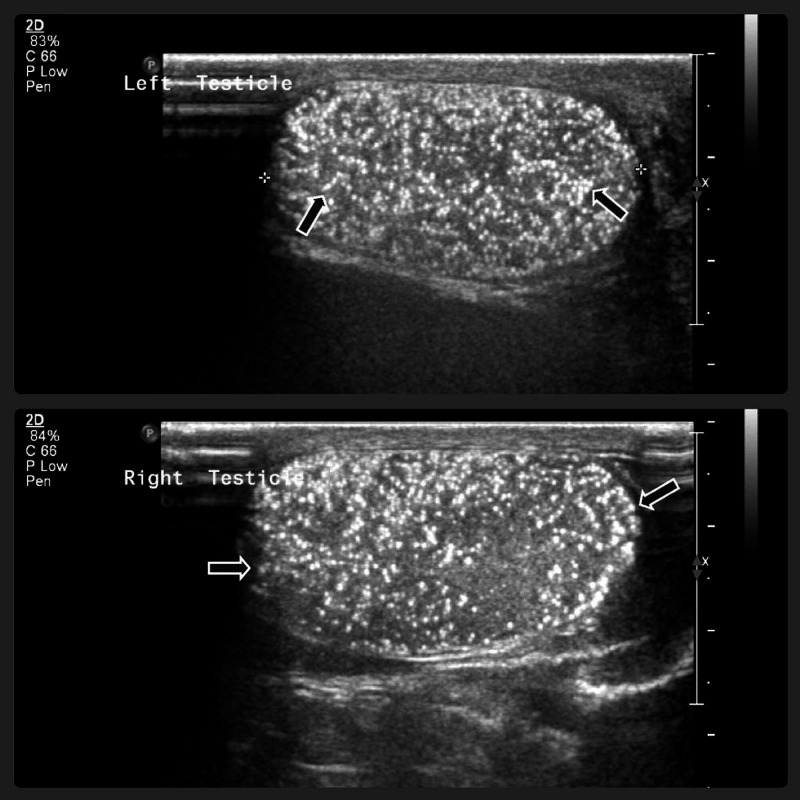
Sonographic appearance of the testes shows diffuse testicular calcifications consistent with microlithiasis.

Computed tomography (CT) results for the abdomen and pelvis were normal. Scrotal US showed bilateral diffuse testicular microcalcification (Grade IV microlithiasis) without a focal lesion (Figure 2).

The serum lactate dehydrogenase (LDH) level was 512 U/L (normal range: 100-190 U/L), β-human chorionic gonadotrophin (β-HCG) level was 52.51 (normal range: 0-6 mIU/mL), and α-fetoprotein (AFP) was 2479.34 IU/ml (normal: 0-9 IU/ml). Other laboratory results, including hepatic and renal panels, were within normal limits. Transthoracic needle biopsy of the mediastinal tumor showed a mixed germ cell tumor. The diagnosis was enhanced by immunohistochemistry: focal positivity for placental alkaline phosphatase (PLAP), negative for neuron-specific enolase (NSE). Semen analysis showed complete asthenozoospermia, oligospermia, and hypospermia (volume, 0.4 ml; count, 0.26 × 106/mL; 100% immotile spermatozoa). Our diagnosis was primary mediastinal nonseminoma germ cell tumor (poor risk) with TM.

Therapeutic intervention

The standard chemotherapy regimen for poor-risk disease is four cycles of bleomycin, etoposide and cisplatin (BEP). Alternatively, four cycles of etoposide, ifosfamide, and cisplatin (VIP) can be used to treat patients who may not tolerate bleomycin. Both regimens are category 1 recommendations (National Comprehensive Cancer Network (NCCN) guidelines, version 1.2021). The patient was treated with four cycles of chemotherapy consisting of VIP.

Follow up and outcomes

**Figure 3:**
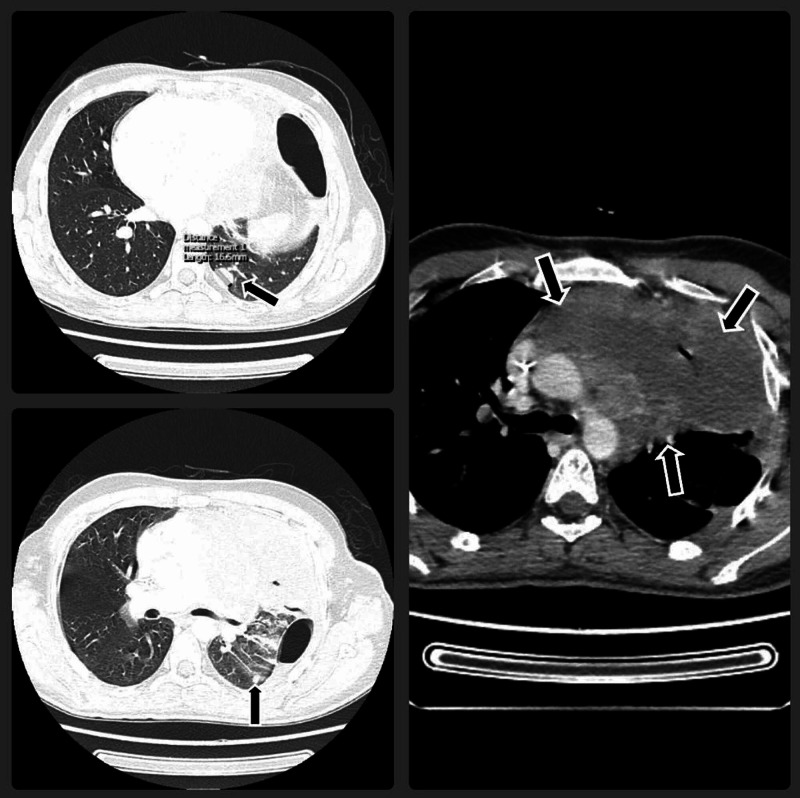
Computed tomography (CT) of the chest following chemotherapy shows residual mass in the superior mediastinum with appearance of two new lung lesions.

Although tumor markers following chemotherapy returned to normal, CT revealed a residual mediastinal mass with the appearance of two new lung lesions (Figure 3).

The patient was then referred to a different oncology center for additional cycles of chemotherapy giving that consultation with a high-volume center should be considered for the management of such a patient.

## Discussion

TM is an uncommon condition in which microcalcifications are present in the seminiferous tubules [4]. These microcalcifications can be seen by US as hyperechoic, nonshadowing foci measuring 1-2 mm in diameter throughout both testes [7]. Few studies to evaluate the prevalence and natural history of TM have been performed. Cast et al. reported a prevalence of 0.68% in 4819 referred patients [8]. 

TM can be associated with cryptorchidism, Klinefelter and Down syndromes, varicocele, testicular torsion, and male pseudohermaphroditism [2,5]. The clinical significance of TM is not completely understood; however, a correlation between TM and testicular cancer has been found [3]. A meta-analysis conducted by Wang et al. suggests that TM is significantly associated with risk of testicular cancer [9]. A correlation between TM and extragonadal germ cell tumor is not well defined and their incidence is rare, with only few reported cases [7,8]. To the author's knowledge, this report presents only the seventh case of extragonadal germ cell tumor with TM.

## Conclusions

In conclusion, the author has reported a rare case, which should elicit discussion on how to screen and treat such patients with extragonadal germ cell tumors and TM. Testicular US follow-up and CT scan of chest and abdomen with serum tumor markers might be considered in patients with incidental findings of TM.
